# Reunification of Object and View-Center Background Information in the Primate Medial Temporal Lobe

**DOI:** 10.3389/fnbeh.2021.756801

**Published:** 2021-12-06

**Authors:** He Chen, Yuji Naya

**Affiliations:** ^1^School of Psychological and Cognitive Sciences, Peking University, Beijing, China; ^2^IDG/McGovern Institute for Brain Research, Peking University, Beijing, China; ^3^Beijing Key Laboratory of Behavioral and Mental Health, Faculty of Science, College of Psychology and Cognitive Sciences, Peking University, Beijing, China

**Keywords:** macaque monkey, medial temporal lobe, perirhinal cortex, inferotemporal cortex, ventral pathway, figure-ground segmentation, relational space, view-center background

## Abstract

Recent work has shown that the medial temporal lobe (MTL), including the hippocampus (HPC) and its surrounding limbic cortices, plays a role in scene perception in addition to episodic memory. The two basic factors of scene perception are the object (“what”) and location (“where”). In this review, we first summarize the anatomical knowledge related to visual inputs to the MTL and physiological studies examining object-related information processed along the ventral pathway briefly. Thereafter, we discuss the space-related information, the processing of which was unclear, presumably because of its multiple aspects and a lack of appropriate task paradigm in contrast to object-related information. Based on recent electrophysiological studies using non-human primates and the existing literature, we proposed the “reunification theory,” which explains brain mechanisms which construct object-location signals at each gaze. In this reunification theory, the ventral pathway signals a large-scale background image of the retina at each gaze position. This view-center background signal reflects the first person’s perspective and specifies the allocentric location in the environment by similarity matching between images. The spatially invariant object signal and view-center background signal, both of which are derived from the same retinal image, are integrated again (i.e., reunification) along the ventral pathway-MTL stream, particularly in the perirhinal cortex. The conjunctive signal, which represents a particular object at a particular location, may play a role in scene perception in the HPC as a key constituent element of an entire scene.

## Introduction

**FIGURE 1:**
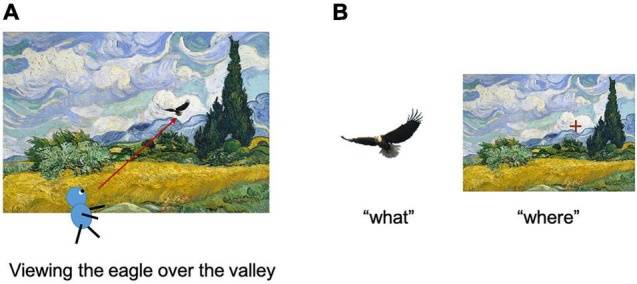
Encoding of items and their location in a scene. **(A)** Assume that a subject is viewing the eagle in the wheat field. **(B)** The item information of the eagle (left) and its location in the environment (right) were acquired using this process. Adapted with permission from Chen and Naya (2020b).

Scene perception is a cognitive function used to construct a mental representation of the external world. The scene construction of primates, including humans, depends on the visual modality, which allows us to know “what” we are currently looking at, and “where” it is in the environment (Figure 1). Traditionally, the two aforementioned types of information are processed in different brain pathways (the two-stream theory) (Mishkin and Ungerleider, 1982; Haxby et al., 1991). The first is called the ventral pathway, which deals with object-related information (“what”), while the other is called the dorsal pathway, which deals with space-related information (“where”). Previously, item encoding in the ventral pathway was considered to be accompanied by a loss of spatial information, such as the retinal position. Although this conventional theory is still dominant in neuroscience research, recent studies have reported that the ventral pathway likely represents space-related information in addition to object-related information (Nowicka and Ringo, 2000; Sobotka et al., 2002; Lehky et al., 2008; Kornblith et al., 2013; Vaziri et al., 2014; Connor and Knierim, 2017). However, researchers are yet to suggest an underlying substance for space-related information in the ventral pathway. The possible reasons for this may include a lack of appropriate task paradigms, which allows us to understand its unique implication distinct from that being extensively investigated in the “where” pathway.

In this review, we first briefly summarize the anatomical projections from the two streams of visual association areas to the medial temporal lobe (MTL). We then list the coding properties of the item signals and recently reported space-related information in the ventral pathway-MTL stream. Finally, we discuss our recent studies that investigated the neuronal representations of object and space-related information along the ventral pathway-MTL stream using a newly devised short-term memory task, called the item-location-retention (ILR) paradigm (Chen and Naya, 2020a,b). According to the preceding literature and our recent studies using the ILR paradigm, we propose a new hypothesis (reunification theory), which includes two conceptual advances. First, in addition to object information, the ventral pathway-MTL stream carries background information on the retina, including parafoveal vision, at each gaze. This view-center background signal could explain most of the space-related information in the ventral pathway-MTL stream reported by previous studies (Georges-François et al., 1999; Kornblith et al., 2013; Vaziri et al., 2014; Hong et al., 2016). Second, information on an object’s position is acquired along the ventral pathway-MTL stream by a constructive process integrating the object and view-center background signals, which are derived from the same retinal image and separately processed along the ventral pathway. The reunification theory may provide a foundation for understanding the scene construction process to support the visual perception, as well as episodic memory.

## Anatomy of the Primate Temporal Lobe

According to the two-stream theory, item- and space-related processing from the primary visual cortex reaches the inferotemporal (IT) cortex (composed of TEO and TE) and posterior parietal cortex, through V4 and the middle temporal area (MT), along the ventral and dorsal pathways in macaques (i.e., occipito-temporal and occipito-parietal paths), respectively. These two pathways connect to the hippocampus (HPC) via the surrounding MTL cortical regions (Albright and Stoner, 2002; Kravitz et al., 2011, 2013).

Along the ventral pathway-MTL stream, the signal in V4 propagates to TEO and succeeds in TE (Distler et al., 1993; Saleem et al., 1993). Subsequently, the signal in TE propagates to the MTL through the PRC and reaches the HPC via the entorhinal cortex (ERC) (i.e., V4-TEO-TE-PRC-ERC-HPC) (Van Hoesen and Pandya, 1975; Suzuki and Amaral, 1994a; Lavenex and Amaral, 2000; Squire et al., 2004). Moreover, V4 connects ventromedially to the posterior subregion of the PHC (TFO) (Ungerleider et al., 2008), which then connects to its anterior subregion (TF/TH) (Suzuki and Amaral, 1994b). The signal in the anterior PHC propagates directly (Suzuki and Amaral, 1994b; Ho and Burwell, 2014) and indirectly (via the PRC) (Suzuki and Amaral, 1994a; Lavenex et al., 2004) to the ERC before the HPC (i.e., V4-PHC-[PRC]-ERC-HPC). It should be noted here that the PHC receives inputs from the early stages of the ventral pathway in addition to those from the dorsal pathway (see below).

Along the dorsal pathway-MTL stream, the signal in the MT propagates to the inferior parietal lobule (IPL) (Rozzi et al., 2006) and enters the MTL directly through the PHC (Cavada and Goldman-Rakic, 1989), or indirectly via the posterior cingulate cortex (PCC) and retrosplenial cortex (RSC) [i.e., MT-IPL-(PCC-RSC)-PHC-ERC-HPC] (Van Hoesen and Pandya, 1975; Vogt and Pandya, 1987; Suzuki and Amaral, 1994b; Morris et al., 1999; Kobayashi and Amaral, 2003, 2007; Kondo et al., 2005).

Separate visual processing of the item and space in the occipito-temporal and occipito-parietal paths in humans was revealed by functional brain imaging and patient studies (Haxby et al., 1991; Jeannerod et al., 1994; Grill-Spector et al., 2001). The connectivity of the ventral pathway (ventrolateral temporal lobe-PRC-HPC) (Kahn et al., 2008; Libby et al., 2012) and dorsal pathway-MTL streams (IPL-PCC-RSC-PHC-HPC) (Rushworth et al., 2006; Kahn et al., 2008; Margulies et al., 2009; Vincent et al., 2010; Libby et al., 2012) in humans is generally comparable with that in macaques (Kravitz et al., 2011, 2013; Ranganath and Ritchey, 2012; Libby et al., 2014). The two-stream theory has also been applied to rodent studies, especially those investigating the MTL system. In consistent with the primates, the rodent PRC-lateral ERC-HPC and postrhinal cortex (rodent homolog of PHC)-medial ERC-HPC circuits have been suggested to process visual items and space information in parallel (Burwell et al., 1995; Burwell and Amaral, 1998; Witter et al., 2000; Eichenbaum, 2006; Furtak et al., 2007).

## Object Coding in the Ventral Pathway

Neurons in the higher visual areas receive inputs from each of the earlier brain areas with smaller receptive fields, either directly or indirectly. After simple algebraic operations in the early visual areas, hierarchically organized information processing realizes neurons displaying comparatively more complex response properties to objects along the ventral pathway (Felleman and Van Essen, 1991). The complexity of neuronal responses reportedly increases with the size of the receptive field in non-human primates. While the receptive field is 0.5–2° near the fovea in V1, it is typically 2–10° in V4. The receptive fields of the IT cortex neurons are enlarged further (10–30°) and substantially cover bilateral portions of the visual field (Hubel and Wiesel, 1968; Kobatake and Tanaka, 1994; Roe et al., 2012). Thus, while V1 neurons distinguish the orientation, spatial position, and movement direction of a small stick, IT cortex neurons respond to a large complex shape, containing multiple visual features, the selectivity of which does not depend on the size of the stimuli or retinal position (Schwartz et al., 1983; Kobatake and Tanaka, 1994).

Rather than a simple pixel-wise representation of the retinal image, we perceive a visual object by supposedly representing its inner spatial configurations, regardless of its actual size and position on the retina (Connor and Knierim, 2017). The transformation from retinal representation to relational representation proceeds sequentially along the anatomical hierarchy of the visual areas. For example, a spatial relationship among the points along an extended contour on the retina is combined to construct orientation tuning in V1. Changes in the orientation (e.g., abrupt for corners and gradual for curves) then construct curvature tuning in V4. After the curvatures are assembled into local configurations (e.g., eyes), their spatial relationship constructs coherent object tuning (e.g., faces) in the IT cortex. Therefore, relational coding is specialized for object processing at the expense of the loss of the absolute retinal position of the perceived object.

## Mnemonic Effects on Object Coding in the Medial Temporal Lobe

While TE is located at the last stage of visual object perception (Miyashita, 1993; Sheinberg and Logothetis, 1997), the PRC is located at the entrance of the MTL and serves as a hub with converging inputs from a wide range of unimodal and polymodal association areas, including TE (Suzuki and Naya, 2014; Miyashita, 2019). A functional double dissociation between these two adjacent brain regions was reported by lesion studies using two distinct tasks requiring sensory perception (e.g., a color discrimination task) and recognition (e.g., delayed non-matching task) (Buckley et al., 1997). While TE is more responsible for visual perception, the PRC is critical for memory functions, such as item recognition (Buffalo et al., 1999; Buckley et al., 2001; Baxter, 2009).

Neurophysiological studies have further revealed different neural operations between the PRC and TE, particularly in the visual pair-association (PA) paradigm (Naya et al., 2001, 2003), which examines the semantic association memory of visual objects (Sakai and Miyashita, 1991). In the PA task, a visual object was presented as a cue stimulus, and after a delay period, monkeys were required to choose a particular visual object that had been assigned as a paired associate of the cue stimulus and should be retrieved from long-term memory. During the PA task, a substantial number of neurons in both the PRC and TE showed item-selective responses to cue stimuli (Naya et al., 2003). Among the item-selective neurons, some neurons showed a correlated response to the visual objects of the same pairs during the cue stimulus presentation (“pair-coding neuron”) (Sakai and Miyashita, 1991). The proportion of pair-coding neurons dramatically increased when the visual signal was transmitted forward, from TE (4.9% of the item-selective neurons) to the PRC (33%) (Naya et al., 2003). In addition to the pair-coding neurons, the PA task revealed a separate group of neurons that represented the to-be-retrieved target, the paired associate of the cue stimulus (“pair-recall neurons”) (Sakai and Miyashita, 1991). The memory retrieval signal appeared in the PRC, even during the cue stimulus presentation (∼200 ms after cue onset), while neurons in TE were gradually recruited to display the retrieved visual objects (∼500 ms) after the emergence of the memory-retrieval signal in the PRC (Naya et al., 2001). These findings suggested backward spreading of the memory-retrieval signal from the PRC to TE, which was supported by a following study simultaneously recording from the PRC and TE (Takeda et al., 2015). Together, the series of neurophysiological studies using the PA paradigm showed that neurons in the PRC contribute to item-item association memory, while those in TE provide the PRC with item signals and receive to-be-retrieved item information from the PRC.

## Scene-Selective Response in the Ventral Pathway-Medial Temporal Lobe Stream

The brain regions responsible for scene processing were explored in human neuroimaging studies, which compared BOLD signals when human subjects viewed scene-like stimuli and object-like stimuli (e.g., face). The scene-selective regions included the human transverse occipital sulcus (TOS), RSC, and parahippocampal place area (PPA) regions (Epstein and Kanwisher, 1998a,b; Epstein et al., 2003, 2008). The aggregated anatomical localization of these regions along the dorsal pathway-MTL stream has led to the conclusion that the dorsal pathway-MTL stream is exclusively involved in scene processing for memory-guided behavior, in particular, spatial navigation (Ranganath and Ritchey, 2012; Epstein et al., 2017).

However, recent electrophysiological studies testing non-human primates as subjects have mentioned that single neurons in the ventral pathway can also code spatial information. For example, by combining electrophysiology with functional magnetic resonance imaging, Kornblith et al. (2013) identified two scene-selective brain regions, referred to as the lateral place patch (LPP) and the medial place patch (MPP). The LPP is located in the occipitotemporal sulcus and corresponded to TEOv, while the MPP is located in the posterior subdivision (TFO) of the PHC. Both regions receive strong connections from V4 (see section “The Anatomy of the Primate Temporal Lobe,” above). According to the location proximity and selectivity to scene-like stimuli, these two brain regions have been suggested as homolog areas of the human PPA, which is located in the PHC (Epstein and Julian, 2013). These findings suggest that the response of the human PPA could be explained by the inputs from the ventral pathway-MTL stream, as well as by inputs from the dorsal pathway-MTL stream.

In addition to the ventral part of the temporal lobe just anterior to V4, another brain region was identified as showing spatial information by Vaziri et al. (2014). In the macaque TEd, the majority of neurons responded strongly to large-scale environmental stimuli, in contrast to the weak response to object-sized stimuli. These scene-selective areas in the macaque temporal lobe (i.e., LPP, MPP, and TEd) receive visual signals directly or indirectly from V4 (see section “The Anatomy of the Primate Temporal Lobe,” above), which is suggested as the beginning point of figure-ground segmentation (Roe et al., 2012). We hypothesize that in addition to a figure, which is processed as an object, the ventral pathway would process a background, which might have been observed as a scene-selective response in previous studies (Epstein and Kanwisher, 1998a; Kornblith et al., 2013; Vaziri et al., 2014). Consistent with this idea, recent rodent studies have reported that the PRC of the ventral pathway-MTL stream contributes to spatial or visual scene processing as well as conventional item processing (Fiorilli et al., 2021; Lee et al., 2021).

## Location in the Scene

In addition to the scene-selective responses, spatial information was observed in the ventral pathway for object position by applying a population decoding method to multi-unit recording (Hong et al., 2016). In this experiment, the subjects kept fixating at a central spot and passively viewed the object stimuli that were sequentially presented at different locations in the visual field. To examine the potential spatial information, the researchers gathered the multi-unit firing rates and conducted population decoding using a linear classifier. The success rate of decoding for the object position was higher in the IT cortex than in V4. Thus, the population decoding results suggest that the spatial information was augmented at the late stage of the visual ventral pathway relative to its early stage, while spatial information could not be detected at the single neuron level.

Similarly, many previous visual neuroscience studies have examined the effect of object position while the subjects’ eye position was kept at a central spot and the stimuli were presented in their peripheral visual field [peripheral-view (P-V) condition design]. These studies reported the spatially invariant object representation at least at the single neuron level (Schwartz et al., 1983; Desimone et al., 1984; Miyashita and Chang, 1988). However, when we look at an object in real life, we usually move our eyes toward it and automatically obtain its location in the surrounding environment.

**FIGURE 2:**
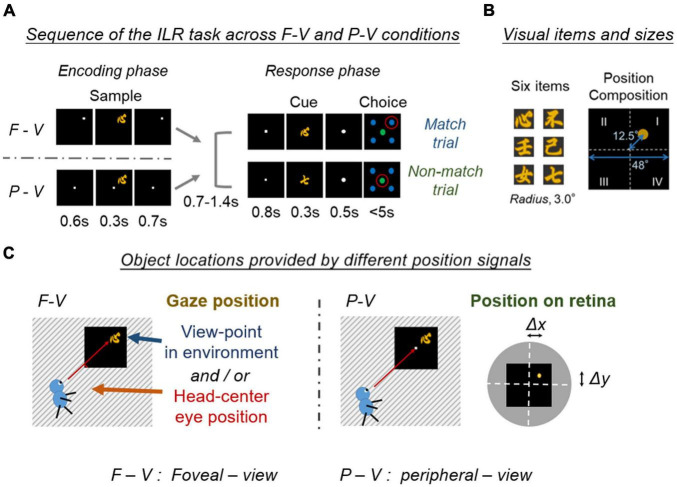
Encoding of the location and item in the two viewing conditions. **(A)** Schematic diagram of the location and item encoding the F-V and P-V conditions of the ILR task. The cue stimulus was the same as the sample stimulus in the match trial (Top), while the two stimuli differed in the non-match trial (Bottom). Red circles indicate the correct answers. Adapted with permission from Chen and Naya (2020b). F-V, foveal-view; P-V, peripheral-view; ILR, item-location-retention. **(B)** Six visual item stimuli, and a spatial composition during the sample period. Adapted with permission from Chen and Naya (2020a). **(C)** Object locations provided by different position signals between the F-V and P-V conditions. The task screen is indicated by a black square and the surrounding environment is indicated by a stripe. The object location was provided by the gaze position in the F-V condition, while the object location was provided by the retinal position in the P-V condition. Note that the gaze position in the F-V condition can be defined by either the eye position in the head-center coordinate or the viewpoint in the environment. The object position on the retina could be defined as a shift from the center of the fovea (Δx, Δy). White dashed lines indicate the horizontal and vertical meridians of the visual field. The yellow dot indicates the center of the sample stimulus. Black square indicates the task screen. The gray disk indicates the surrounding environment in the visual field.

To understand the possible neural patterns closer to everyday behavior, Chen and Naya (2020a; 2020b) adopted a foveal-view (F-V) condition design for the ILR task, which required subjects to encode the identity of a sample stimulus object and its location in each trial (Figure 2). In the F-V condition, subjects fixated on a white square presented within one of the four quadrants of a display. After fixation, one of the six visual objects was presented in the same quadrant as the sample stimulus. After this encoding phase, the response phase was initiated with a fixation dot presented at the center of the screen. One of the visual objects was then presented at the center as a cue stimulus. When the cue stimulus was the same as the sample stimulus, the subject was required to manually answer the sample position (i.e., match trial). Otherwise, the subject was required to choose the disk in the center, regardless of the sample position (i.e., non-match trial). Thus, the ILR task required subjects to encode and retain the identity and location of a sample object stimulus.

**FIGURE 3:**
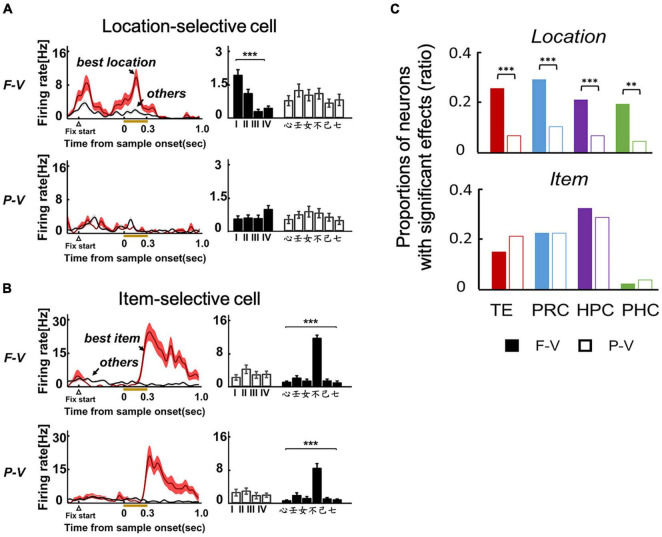
Location and item selective responses in the two viewing conditions of the ILR task. **(A)** Example of a location-selective cell from TE in the F-V (Top) and P-V (Bottom) conditions of the ILR task. The neuron showed location-selective responses only in the F-V condition. **(B)** Example of an item-selective cell from TE. The neuron showed the same preferred item between the F-V and P-V conditions. ***P < 0.0001. Two-way ANOVA with interaction for each cell and view condition. **(C)** Proportions of location-selective cells (Top) and item-selective cells (Bottom) during the sample period in the F-V (Filled bars) and P-V (Open bars) conditions in the ILR task. **P = 0.0011, X^2^ = 13.24, d.f. = 1 for PHC. ***P < 0.0001, X^2^ = 20.67, 20.01, and 30.82 for TE, PRC, and HPC, respectively. *P* values were corrected by Bonferroni corrections among the four recording regions. Adapted with permission from Chen and Naya (2020b).

A substantial number of neurons (20–30%) exhibited location-selective responses in TE of the ventral pathway (Figure 3A), as well as the PRC, HPC, and PHC of the MTL in the F-V condition of the ILR task (Figure 3C, top). Neurons in these brain areas, except for the PHC, also showed item-selective responses (Figures 3B,C, bottom). The selectivity to the location and item was also examined in the P-V condition, in which the subjects maintained fixation to the center spot and the sample object was presented in the peripheral visual field (Figure 2). Although the ILR task required the subjects to acquire the same task-relevant information (e.g., “心” and “Position I”) for the following response in both view conditions, the location-selective responses were substantially diminished in the P-V condition for TE (Figure 3A) and the MTL areas (Figure 3C, top). These results indicate that the ventral pathway-MTL stream signals an object position even at the single neuron level when subjects look at an object by their foveal vision, because neurons exhibit robust activities that are selective to gaze positions. In contrast, item selectivity did not differ between the two view conditions (Figures 3B,C, bottom), confirming the spatially invariant representation of object information.

**FIGURE 4:**
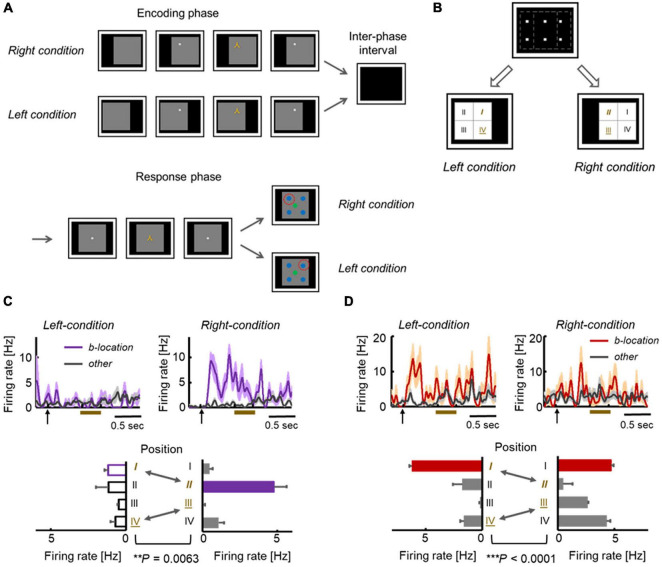
Location-selective responses in conditional-type ILR Task. **(A)** Sequences in the conditional-type ILR task showing the left and right conditions. The time parameters are the same as those in the standard ILR task, except for the initial presentation of the gray square (0.5 s). **(B)** Spatial configuration of the stimulus positions in the left and right conditions. The first and fourth quadrants in the left condition (“I” and “IV”) were also used as the second and third quadrants in the right condition (“II” and “III”). **(C)** Example of an HPC neuron in which responses to the same fixation position differed between the two conditions. **(D)** Example of a TE neuron showing significantly different responses between the two conditions. Adapted with permission from Chen and Naya (2020a).

The gaze-dependent activity could be explained by the following factors (Figure 2C). One type of factor is derived from the somatosensory (proprioception) and motor systems, which are directly related to the internal control of eye movements. The other type is derived from the visual system, which provides information on a retinal image at each gaze position. To dissociate the two types of gaze-related effects, additional experiments were conducted by modifying the F-V condition of the ILR task (i.e., conditional-type ILR task). In the modified version of this task, a large gray square was first presented on the right or left side of the display. Subsequently, the fixation dot and sample stimulus were presented sequentially during the encoding phase in the same way as in the F-V condition (Figures 4A,B). After the inter-phase interval with a blank screen, a large gray square was presented in the center of the display in both right and left encoding conditions, and the subject was required to answer the sample position relative to the large gray square in the match trials.

**FIGURE 5:**
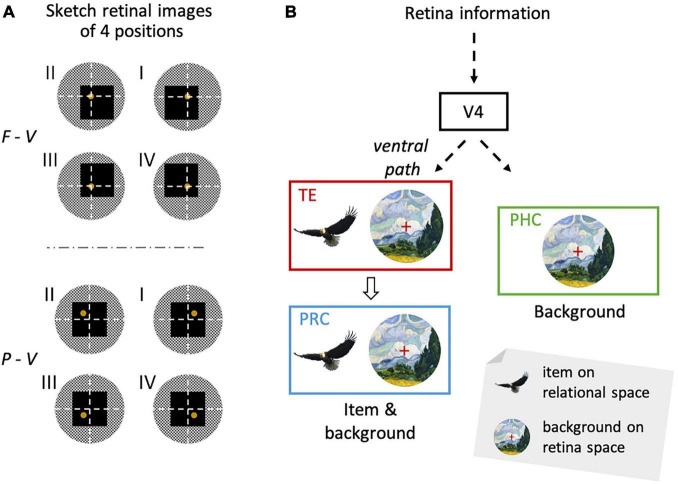
Location signal reflecting background information and the item signal from the same retinal image. **(A)** Schematic diagram of visual inputs to the retinae during the sample period. White dashed lines indicate the horizontal and vertical meridians of the visual field. Yellow disks indicate the sample stimuli (i.e., yellow Chinese characters). Black squares indicate the task screen. Gray circles indicate the surrounding environment. Please note the larger change in the retinal images across different quadrants of the sample stimulus in the F-V condition compared with the P-V conditions. **(B)** The visual information from the retina reaches TE through the ventral pathway. In TE, there are not only item signals insensitive to the retinal position information, but also location signals provided by the background information sensitive to the retinal position information. The information has then been transferred to the PRC. While the PRC has two types of signals, the PHC has only the location signal. Adapted with permission from Chen and Naya (2020b). F-V, foveal-view; P-V, peripheral-view; ILR, item-location-retention.

Across TE and the MTL areas, neurons do not necessarily represent the gaze positions themselves. For example, Figure 4C shows an HPC neuron that exhibited location-selective activity only in the right condition. This neuron exhibited the strongest response to the second quadrant in the right condition. However, the same neuron showed only negligible responses to the first quadrant in the left condition, although the two were at the same positions in the head-center coordinates (Figure 4B). Some other neurons showed preferred responses to the same quadrant in both the left and right conditions (Figure 4D). In short, the responses of these neurons were related with the visual inputs, rather than the head-center gaze positions. As shown by these examples, the location selective activity in the F-V condition could not be explained by the gaze positions themselves. Presumably, neurons in the primate temporal lobe signal the retinal image reflecting background information (including the parafoveal vision), which would specify the current gaze position and object position in the F-V condition (Figure 5A, top). The overall shift in the retinal images across the different gaze positions would result in a substantial number of neurons exhibiting location-selective activity in the F-V condition, while the local change of the retinal image across the different sample positions would allow only a small number of neurons to show location-selective activity in the P-V condition (Figures 3C, 5A). We refer to the information of the retinal image specifying gaze position as the “view-center background signal.” Previous studies have elucidated the effect of eye position in the ventral pathway (Nowicka and Ringo, 2000; Lehky et al., 2008). Interestingly, Norwicka and Ringo revealed the effect of eye position on the responses of IT neurons under both light and dark conditions. However, separate neural populations exhibited eye position-sensitive responses between the two conditions (i.e., the presence and absence of visual inputs). Therefore, some IT neurons could be driven by eye-position-relevant inputs from non-visual modalities, but the visual input may be dominant in the primate IT cortex and their eye position selective responses could be explained by the view-center background, at least when it is available (i.e., light condition).

To examine the task dependence of the view-center background signal, Chen and Naya (2020a) performed another independent experiment free of memory demand using a passive-encoding task (Chen and Naya, 2020a). They found that the view-center background signal, which is accompanied by gaze behavior, is automatically encoded into the MTL along the ventral pathway. In contrast, the item signal was not detected in the passive-encoding task, regardless of the viewing conditions. The loss of item-selective response can presumably be attributed to a combination of the features of man-made complex stimuli (i.e., Chinese characters) and the passive-encoding task, which would not allow the sample stimulus to be segmented from the background as an object.

The view-center background signal is necessarily an egocentric spatial representation but may also provide us with allocentric spatial information of a target location in an environment. Rolls (1999) previously reported the HPC “view cells,” which exhibited selective responses to a particular target location where a monkey gazed. This view-location-selective response was not sensitive to the monkey’s self-position in a room, and consequently could not be explained by the gaze position itself. It can be assumed that the location-selective responses of view cells could be explained by the view-center background signal for the following reasons: the view-center background of a particular target location would be relatively similar across different self-positions compared with that of a different target location. For example, the retinal image changes according to the distance and direction between the retina and light sources, which depend on the subject’s movement, but the relationship among segmental images of the neighboring retinal positions is generally maintained as long as the subject views the same target location. In contrast, different target view locations would produce different view-center background signals. Thus, the existence of a view-center background signal reconciles the first person’s perspective with the allocentric representation of the space.

The view-center background signal in the ventral pathway is considered as one aspect of space-related information, while the dorsal pathway is related with other aspects of space-related information such as a direction of gaze, a position with respect to head and a self-location in an environment (Olson et al., 1996; Ranganath and Ritchey, 2012). The different aspects of spatial processing in the ventral and dorsal pathways may require a modification of the “two-stream theory” but still acknowledge it (Goodale and Milner, 1992, 2018).

## Reunification of Item and View-Center Background Signals

**FIGURE 6:**
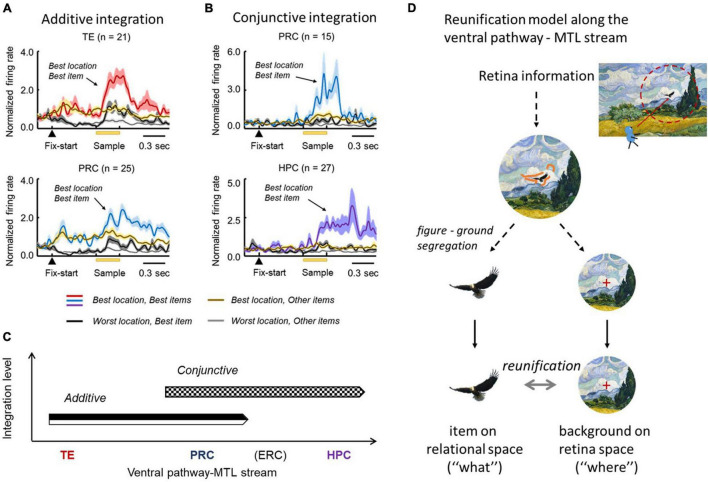
Stepwise linkage of the location and item signals along the TE-PRC-HPC stream. **(A)** Population-averaged responses of neurons displaying additive integration in TE and the PRC in the F-V condition of the ILR task. These neurons started the location-selective responses after fixation, and the item-selective response was added after the sample presentation. **(B)** Population-averaged responses of neurons displaying conjunctive integration. Please note that the responses in the best location and best item demonstrate a robust increase, following the sample presentation in both the PRC and HPC. Adapted with permission from Chen and Naya (2020a). F-V, foveal-view; ILR, item-location-retention. **(C)** Additive and conjunctive types of integration process occurred sequentially along the ventral pathway-MTL stream. **(D)** Reunification model. Through the ventral pathway, the visual information from the retina first went through the figure-ground segregation process, where the item and location signals were segregated. After the two signals were separately processed, they were integrated again (i.e., “reunification”), at the single-unit level. The reunification process may be supported by the additive and conjunctive integrations along the ventral pathway—MTL stream.

In the F-V condition of the ILR task, the co-existence of item and view-center background signals (Figures 3C, 5B) prompted the ventral pathway-MTL stream to integrate them at the single neuron level. In TE and the PRC, there were significantly larger numbers of neurons that exhibited both signals in an additive manner (Figure 6A). There were also other types of integration neurons in the PRC and HPC (Figure 6B). The second type of neuron exhibited activities selective to both the item and location of a sample object, only after the stimulus was presented. In other words, this type of integration neuron did not show location-selective activity before the sample stimulus presentation, even though the subjects gazed at a position where a sample stimulus would be presented. The neurons represented a combination of the identity and location of the sample stimulus rather than the two signals.

The two types of integrations proceeded sequentially along the ventral pathway-MTL stream (Figure 6C). Here, we propose a new theory that explains the neural mechanisms that encode the identity and location of an object in a scene (Figure 6D). After figure-ground segmentation, which reportedly occurs at V4 (Roe et al., 2012), the object information and background information are separately processed along the ventral pathway, and the two signals are then reunified (“reunification model”) to signal a particular item at a particular location. The representations of the object-related information and the space-related information by different neural ensembles in the reunification model contrast with the population coding which has rapidly prevailed in computational neuroscience, particularly after the invention of “deep learning” (Hong et al., 2016). Along the ventral pathway-MTL stream, the PRC may play a key role in the integration process to develop an additive representation into the conjunctive representation. We hypothesize that the object-location signal carried by the PRC conjunctive integration neurons would be transmitted to the HPC, in which additional association would proceed. The prominent integration effect of the PRC for perceptual processing may underlie the formation of long-term association memory in the PRC (see section “Mnemonic Effects on Object Coding in the MTL” above) (Suzuki and Naya, 2014).

In contrast to the reunification model for the object location relative to the background, Aggelopoulos and Rolls (2005) suggested that neurons in the macaque IT cortex encoded object locations relative to other objects presented at once in a passive fixation task. They reported that receptive fields of the IT neurons were reduced into the center of visual field as well as became more asymmetric in the presence of multiple objects compared with a condition when a single object was presented by itself. The reduced size of receptive fields may be explained by mutual inhibitions among neurons that were excited by the neighboring objects on the retinae (Aggelopoulos and Rolls, 2005) and/or by a lack of selective attentions to individual objects due to the competitions among the objects, which may influence the figure-ground segmentation. The processing of object information on the relational space might be an active process depending on the selective attention to a target either voluntarily (e.g., ILR task) or involuntarily (e.g., passive-encoding task with a single object). It would be useful to test the reunification model in a complex natural scene containing multiple objects in future studies.

## Scene Construction

The reunification model explains the perception or encoding of an object and space in a single snapshot of view. In each separate view, the TE-PRC-HPC stream provides the HPC with a conjunctive signal. However, multiple saccades are necessary to construct an entire scene in daily life. The across-saccades coordination might be mediated by the PHC, which receives inputs from the dorsal pathway and shows modulations of neuronal activity by eye-position/movement (Olson et al., 1996). We hypothesize that multiple shots of view-center backgrounds would be combined across saccades along the dorsal pathway-MTL stream. Furthermore, the combined space representation is integrated with the conjunctive signal from the TE-PRC-HPC stream in the HPC to construct a coherent scene, including objects from the first person’s perspective on the perception and encoding of episodic memory (Baxter, 2009; Suzuki, 2009), which would contribute to a subsequent recollection-based recognition (Eichenbaum et al., 2007). Eventually, an intrinsic relationship among the multiple snapshots of views may also serve as an allocentric cognitive map in the HPC, which has often been reported in the context of spatial navigation that requires information about the environment (Vogt et al., 1992; Iaria et al., 2007; Burgess, 2008; Epstein, 2008; Kravitz et al., 2011; Zhang and Naya, 2020).

## Conclusion

In contrast to the conventional two-stream theory, the ventral pathway carries as much space-related information as object-related information. The space-related information is likely substantiated by a large background image projected onto the retina. This view-center background image may not only provide the first person’s perspective, but also specify a viewing location in an environment to provide allocentric spatial information. The object signal and the view-center background signal are transmitted along the ventral pathway-PRC-(ERC)-HPC stream when a subject is looking at an object by foveal vision. The two signals are integrated step-by-step to represent a particular object at a particular location along the stream, particularly in the PRC. We refer to this neural model as the “reunification theory” because the two signals are derived from the same retinal image. The object-location information based on the view-center background signal in the ventral pathway-MTL stream is more consistent with the distinction of the ventral/dorsal pathways based on their corresponding behavioral functions (“perception” and “action”) (Goodale and Milner, 1992, 2018) than with that based on their typical representational contents (“what” and “where”) (Mishkin and Ungerleider, 1982; Haxby et al., 1991). Accordingly, the reunification theory does not contradict but specifies the signals in the two-stream theory. Although the reunification theory can explain the perceptual process for a single snapshot of view, multiple saccades are required to perceive the entire scene. We hypothesized that the PHC could combine multiple view-center background images across saccades because it receives the space-related information from the dorsal pathway in addition to the view-center background signal. Future studies should investigate the neural mechanisms responsible for the construction of the entire background in the PHC, as well as for the construction of the entire scene, including objects in the HPC, which would support scene perception and episodic memory.

## Author Contributions

HC and YN wrote the manuscript. Both authors contributed to the article and approved the submitted version.

## Conflict of Interest

The authors declare that the research was conducted in the absence of any commercial or financial relationships that could be construed as a potential conflict of interest.

## Publisher’s Note

All claims expressed in this article are solely those of the authors and do not necessarily represent those of their affiliated organizations, or those of the publisher, the editors and the reviewers. Any product that may be evaluated in this article, or claim that may be made by its manufacturer, is not guaranteed or endorsed by the publisher.
